# Effect of solid-state fermentation on kidney bean flour: Functional properties, mineral bioavailability, and product formulation

**DOI:** 10.1016/j.fochx.2025.102339

**Published:** 2025-03-06

**Authors:** Nikhil Dnyaneshwar Patil, Aarti Bains, Gulden Goksen, Nemat Ali, Sanju Bala Dhull, Mohammad Rashid Khan, Prince Chawla

**Affiliations:** a Department of Food Technology and Nutrition, Lovely Professional University, Phagwara, Punjab 144411, India; b Department of Microbiology, Lovely Professional University, Phagwara 144411, Punjab, India; c Department of Food Technology, Vocational School of Technical Sciences at Mersin Tarsus Organized Industrial Zone, Tarsus University, 33100, Mersin, Turkey; d Département of Pharmacologies and Toxicology, Collège of Pharmacy, King Saud University, Riyadh 11451, Saudi Arabia; e Department of Food Science and Technology, Chaudhary Devi Lal University, Sirsa 125055, Haryana, India

**Keywords:** Fermentation, Functional properties, Kidney bean flour, Mineral bioavailability, thermal stability

## Abstract

This research investigated the effects of solid-state fermentation with *Aspergillus awamori* (MTCC 548) on the mineral bioavailability, structural characteristics, and functional attributes of kidney bean flour across different fermentation durations (24–96 h). Notable improvements were observed during 96 h of fermentation, including significant increases in foaming capacity and stability (by 32.30 % and 34.81 %), emulsifying capacity and stability (by 32.67 % and 47.37 %), oil and water holding capacities (by 72.86 % and 61.87 %, respectively). Bulk density decreased by 48.68 %. Fermented samples demonstrated structural changes and chemical alterations with increased thermal stability at 24 and 48 h, which declined with extended fermentation. The iron and zinc contents increased by 5.95 % and 13.59 %, respectively, after 24 h, with bioavailability improving by 34.53 % and 36.30 %. Additionally, the fermented kidney bean flour enhanced the *in-vitro* digestibility of biscuits by 31.33 %. This study highlights the potential of solid-state fermentation to enhance the nutritional and functional properties of kidney bean flour.

## Highlights

• Fermentation significantly improved the functional attributes of kidney bean flour.
• 24 h of fermentation enhanced the thermal stability of kidney bean flour.
• Fermentation for 72 h increased the mineral bioavailability of the flour.
• Fermentation enhanced the *in-vitro* digestibility of formulated biscuits.

## Graphical abstract

**Figure:**
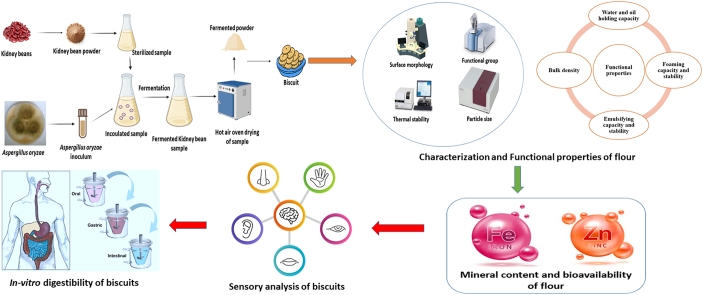

## 1 Introduction

In recent years, consumer preferences have increasingly shifted toward plant-based diets and health-focused snack options. This trend is attributed to the increasing awareness of the health benefits associated with plant-based foods, including their potential to mitigate the risk of chronic diseases, support effective weight management, and promote overall health and well-being (Alae-Carew et al., 2022). Furthermore, growing concerns regarding environmental sustainability and animal welfare have driven a considerable number of individuals to reduce their consumption of animal-derived products and adopt plant-based alternatives. As a result, the plant-based food sector, particularly in the snack category, has witnessed substantial growth, marked by an increasing demand for nutrient-dense and minimally processed options (Kim & Zailani, 2024).

Among the various plant-based alternatives, legumes have traditionally been a staple due to their high protein content, making them an essential food source in many cultures worldwide. Besides protein, legumes are also rich in dietary fiber, essential vitamins, and minerals, offering numerous health benefits such as improved digestion, better glycemic control, reduced cholesterol levels, enhanced cardiovascular health, and a lower risk of chronic diseases (Oz et al., 2023; Sonta & Rekiel, 2020). Among legumes, kidney beans (*Phaseolus vulgaris*) are the most widely consumed variety worldwide. These kidney beans are not only a staple food in various regions but also an important agricultural crop. Major producers of kidney beans include India, Brazil, the United States, and China, which contribute significantly to global production (S. Kumar et al., 2013). From a nutritional perspective, kidney beans (*Phaseolus vulgaris*) are considered due to their high content of protein, dietary fiber carbohydrates, and essential micronutrients, including iron, magnesium, and folate. This dense nutrient profile makes them a valuable component of a balanced diet (Sara et al., 2021). The consumption of kidney beans has been associated with several health benefits, including improved digestion, regulation of blood glucose levels, and a reduction in the risk of chronic diseases such as cardiovascular disease and diabetes (Migliozzi et al., 2015). Additionally, the high dietary fiber content of kidney beans plays a crucial role in weight management and supports gastrointestinal health (Wang et al., 2020). Despite their nutritional benefits, kidney bean flour exhibits limited functional properties, including low water and oil absorption capacities, poor emulsification, and undesirable characteristics such as thermal stability and *in-vitro* digestibility, all of which restrict its direct application in various food formulations (Popoola et al., 2023).

To mitigate the challenges associated with limited functional properties and mineral bioavailability in legumes, solid-state fermentation (SSF) presents a promising approach. SSF involves the cultivation of microorganisms on solid substrates with low or no liquid content, a process that can enhance the nutritional composition and functional attributes of legumes, thereby improving their suitability for various food applications (J. Wang et al., 2023). *Aspergillus awamori,* a key microorganism utilized in SSF, is a filamentous fungus recognized for its role in traditional Asian fermentations, such as soy sauce, miso, and sake. *Aspergillus awamori* secretes a range of enzymes, including proteases, amylases, and phytases, which catalyze the breakdown of complex macromolecules into simpler, more bioavailable forms, thereby improving the nutritional and functional properties of legumes (Cebrián & Ibarruri, 2023). Its safety and utility have been evaluated and approved by various regulatory authorities worldwide. In the United States, specific strains of *A. awamori* have been granted Generally Recognized as Safe (GRAS) status by the FDA for enzyme production (Food and Drug Administration, (FDA), 2025) and similarly, the Food Safety and Standards Authority of India (FSSAI) has also recognized its safety for use in food-related applications (Food Safety and Standards Authority of India (FSSAI), 2025). The literature highlights the versatility of *A. awamori* in enhancing the nutritional profiles of various flours through fermentation. For instance, a study on the solid-state fermentation of lentil flour demonstrated significant improvements in antioxidant profiles, including enhanced total phenolic content, increased formation of bioactive phenolic compounds, and improved mineral bioavailability, showcasing its potential to boost nutritional value (Dhull et al., 2020). Similarly, the fermentation of soybean-defatted flour resulted in mobilizing bioactive compounds and improved *in-vitro* and *in-vivo* antioxidant activity (Georgetti et al., 2009)**.** Additionally, mango kernel flour treated with *A. awamori* and *A. oryzae* showcased enhanced nutritional properties, including reduced anti-nutritional factors, increased protein and fat content, and the presence of prebiotic compounds, indicating its potential as a functional food ingredient and a substrate for beneficial microbial growth (Arnau & Villasante, 2024). *Aspergillus awamori* shows great promise in enhancing the nutritional quality of plant-based ingredients, improving bioavailability, and reducing antinutritional factors. Its potential for developing functional foods with added health benefits makes it a valuable tool for future food innovations.

To date, there has been limited research on the functional properties, mineral bioavailability, product formulation, and *in-vitro* digestibility of kidney bean flour following solid-state fermentation. Therefore, this study was designed to assess the functional properties of solid-state fermented kidney bean flour, evaluate the *in-vitro* bioavailability of zinc and iron, and characterize kidney bean flour. Through this research, we aim to advance the development of more nutritious and health-enhancing legume-based foods.

## 2 Materials and methods

### 2.1 Materials

Kidney bean seeds were sourced from Punjab University, Ludhiana, Punjab, while *Aspergillus awamori* MTCC 548 was purchased from Microbial Type Culture Collection and Gene Bank, Institute of Microbial Technology, Chandigarh, India. Class ‘A' certified glassware, which had been acid-washed, was used throughout the experiments. All reagents and standard solutions were prepared with deionized water.

### 2.2 Methods

#### 2.2.1 Preparation of kidney bean flour

Whole kidney bean seeds were finely ground into flour using a Vitamix Professional Series 750 blender from the United States. The resulting flour was kept in sealed containers at a temperature range of 4–7 °C for future use (Chawla, Bhandari, et al., 2017).

#### 2.2.2 Fermentation of kidney bean flour

##### 2.2.2.1 Strain selection

In this study, the GRAS-classified fungal strain *Aspergillus awamori* MTCC 548 was utilized for solid-state fermentation (SSF) of kidney bean flour due to its superior enzymatic activity and proven ability to enhance the nutritional and functional properties of legumes. Known for producing proteases, amylases, and phytase, this strain improves protein digestibility and enhances mineral bioavailability (Chang et al., 2023; Chawla, Chawla, et al., 2017; Dhull et al., 2020; Senanayake et al., 2020).

##### 2.2.2.2 Media, strain growth conditions, and preparation

The method described by Dhull et al. (2020) was adapted for SSF of kidney bean flour. The fungal culture of *Aspergillus awamori*, maintained on potato dextrose agar (PDA) slants was transferred to freshly prepared PDA plates before the start of each experiment. PDA, a nutrient-rich medium, is widely recognized for supporting the growth and sporulation of filamentous fungi due to its composition, which includes starch from potato infusion and dextrose. These components provide essential nutrients and an accessible energy source, making PDA suitable for fungal cultivation (Borah et al., 2020; Chawla, Bhandari, et al., 2017). The inoculated plates were incubated at 30 °C for 96 h. A spore suspension was prepared using sterile distilled water, ensuring a spore concentration of approximately 1 × 10^6^ spores/ml. Kidney beans were washed thoroughly with deionized water to remove dust and impurities and then dried in a hot air oven. The dried kidney beans were ground to a suitable size for fermentation. 50 g of the kidney bean flour sample were placed into 500 ml Erlenmeyer flasks and soaked overnight at room temperature in 150 ml of Czapek-Dox medium, containing NaNO₃ (2.5 g/L), KH₂PO₄ (1.0 g/L), KCl (0.5140 g/L), and MgSO₄.2H₂O (0.5 g/L). After soaking, the visible droplets of the medium were decanted, and the substrate was autoclaved (121 °C for 15 min) followed by cooling before inoculation. The sterile substrate was inoculated with 5 ml of spore suspension (1 × 10^6^ spores/ml) of *A. awamori*, mixed thoroughly, and incubated at 30 °C for 0, 24, 48, 72 and 96 h. A non-fermented control was prepared by excluding the addition of the spore suspension. At 24 h intervals, fermented samples were collected, tray dried at 45 °C, ground into flour using an electric grinder, and stored at 4 °C for subsequent analysis.

##### 2.2.2.3 Growth determination

Liquid samples were taken at the starting point (0 h), 5 min post-inoculation, and at 24, 48, 72, and 96 h following the inoculation of the kidney bean flour. Viable cell counts were determined on PDA plates using a series of dilutions, following the procedure described in section 2.2.2.2.

##### 2.2.2.4 Determination of pH

The pH levels were monitored every hour throughout the 24, 48, 72, and 96 h fermentation phases using a pH meter (Labman Scientific instrument, UK). A separate bottle, kept under the same conditions, was used to measure the pH of the microorganisms.

#### 2.2.3 Characterization of fermented and non-fermented kidney bean flour

##### 2.2.3.1 Scanning electron microscope (SEM)

The morphological structure of the sample was examined with a SEM, model JCM-6000SEM, from Jeol, Japan. A 5 mg sample was mounted onto carbon-coated copper tape and subsequently sputter-coated for 2 min at a current of 30 mA. Conductivity increased by factors of 170, 350, and 500 when applying accelerating voltages of 15 kV and 20 kV. The images were captured with a working distance set between 8 and 9 mm.

##### 2.2.3.2 Fourier-transform infrared spectroscopy (FTIR)

The functional groups in the powder were analyzed using an FTIR spectrometer (Perkin Elmer X400). 10 mg of the powder was blended with 100 mg of KBr, then inserted into the instrument and covered with a lens for analysis. The data were acquired using Spectrum 10 software, covering the range from 4000 to 400 cm^−1^, with air serving as the background reference.

##### 2.2.3.3 Thermogravimetric analysis (TGA)

Thermogravimetric analysis was employed to evaluate the thermal stability and mass loss of the powders (PerkinElmer Pyris 1 TGA, USA). A 5 mg sample was subjected to a temperature increase from 10 °C to 650 °C at a controlled rate of 10 °C per min, with nitrogen gas flowing at 50 ml per min. Mass changes were monitored throughout the process to assess the decomposition characteristics.

##### 2.2.3.4 Particle size and zeta potential (PA &ZP)

The particle size and zeta potential of powder were analyzed at 24 °C using a Malvern analytical Zetasizer Nano ZS in the United Kingdom. Each powder sample was prepared by mixing the powder with a 1 % solution and then treated with ultrasonication for a duration of 10 min. To ensure precision, measurements were performed in triplicate.

#### 2.2.4 Functional and nutritional properties of fermented and non-fermented kidney bean powder

##### 2.2.4.1 Protein content

The protein content of the fermented and non-fermented flour was analyzed using the Kjeldahl method following the protocol of AOAC (2005).

##### 2.2.4.2 Bulk density (BD)

BD was assessed following the method described by (Ofori et al., 2020). To determine the bulk density, both raw and fermented kidney bean flour samples were precisely introduced into a 10 ml graduated cylinder. The cylinder was gently tapped five times to ensure the contents were settled, and the volume was noted at the 10 ml mark. The bulk density was subsequently calculated by dividing the mass of the powder by its volume (g/cm^3^).

##### 2.2.4.3 Water and oil holding capacity (WHC and OHC)

The WHC and OHC of the flour were evaluated using the procedure outlined by (Patil et al., 2024). To determine water and oil absorption capacities, one gram of each sample was mixed with 15 ml of deionized water or soybean oil in centrifuge tubes that had been pre-weighed. Following a 30-min incubation period, the tubes were subjected to centrifugation at a force of 6000 ×*g* for a duration of 10 min. The supernatant was then carefully removed and discarded. The tubes were weighed again to calculate the absorption capacities, expressed as grams of water or oil retained per gram of sample.

##### 2.2.4.4 Foaming and emulsion properties

###### 2.2.4.4.1 Foaming capacity and stability (FC and FS)

The FC and FS samples were evaluated according to the method described by (Sharma et al., 2023). An initial amount of 0.5 g of flour was blended with 25 ml of deionized water and continuously stirred for 30 min. The mixture was then poured into a 250 ml graduated glass cylinder, and the volume change was recorded to calculate foaming capacity and stability using Eqs. (1), (2), respectively.

(1) $$\text{Foaming capacity}\;\left(\%\right)=\left[\left(\frac{\text{Post whipping volume}\;\left(\mathrm{mL}\right)-\mathrm{Pre}\;\text{whipping volume}\;\left(\mathrm{mL}\right)}{\mathrm{Pre}\;\text{whipping volume}\;\left(\mathrm{mL}\right)}\right)\times 100\right]$$

Foam stability (FS) was analyzed by measuring the volume of foam preserved after incubation for 30 min at a temperature of 25 °C ± 2 °C. The results were presented as a percentage of the foam volume measured initially.

(2) $$\text{Foaming stability}\;\left(\%\right)=\left[\left(\frac{\text{Volume after standing time}\;\left(\mathrm{mL}\right)-\text{Volume before whipping}\;\left(\mathrm{mL}\right)}{\text{Volume before whipping}\;\left(\mathrm{mL}\right)}\right)\times 100\right]$$

###### 2.2.4.4.2 Emulsion capacity and stability (EC and ES)

The EC and ES of kidney bean flour were evaluated using the method detailed by (Sharma et al., 2024). A 1 % *w*/*v* solution of the flour was prepared to serve as the aqueous phase, after which 1 ml of canola oil was added. The solution was stirred magnetically at 5000 rpm for an hour, which resulted in a creamy emulsion. After this, the emulsion was centrifuged at 10,000 ×*g* for 20 min. EC was assessed by recording the height of the emulsified sample within a graduated glass measuring cylinder. The emulsion capacity and stability of the sample were calculated using eqs. (3), (4), respectively.

(3) $$\text{Emulsion capacity}\;\left(\%\right)=\left\{\left(\frac{\text{Volume of the emulsion layer}\;}{\text{Total height of content in the tube}}\right)\times 100\right\}$$

Emulsion stability was evaluated by heating the emulsion to 70 °C for 30 min, followed by centrifugation at 6000 ×*g* for 15 min.

(4) $$\text{Emulsion stability}\;\left(\%\right)=\left\{\left(\frac{\;\text{height of the Emulsified layer after heating}}{\text{height of the emulsified layer before heating}\;}\right)\times 100\right\}$$

##### 2.2.4.5 Mineral content

The zinc and iron levels in both fermented and unfermented samples were determined using an Atomic Absorption Spectrophotometer (AAS) (Model AA-7000, PerkinElmer, Waltham, MA, USA) following the AOAC (2005) guidelines. The samples were ashed at 550 °C for 8 h, followed by solubilization in a tri-acid mixture and heating until fully dissolved. Before analysis by AAS, all samples were diluted to an appropriate concentration (Chawla, Chawla, et al., 2017).

##### 2.2.4.6 Mineral bioavailability

The percentage bioavailability of flour was determined, by simulating the gastrointestinal digestion process and utilizing the Caco-2 cell lines approach specified by (Patil et al., 2023)**.** The cell culture procedures were conducted following the guidelines reported by (Patil et al., 2023)**.**

#### 2.2.5 Preparation of fermented flour incorporated biscuits

The biscuits were prepared following the methodology described by Jindal et al. (2024). The ingredients and their respective quantities for both control and fermented flour-incorporated biscuits are presented in Table 1. Control biscuits (B0) served as the reference for evaluating the biscuits containing fermented flour. A modified version of the method by Widodo and Sirajuddin (2019) was used for preparation. Butter (65 g), vanilla essence (5 ml), and sugar (70 g) were whisked for 2–3 min until a fluffy consistency was achieved. Subsequently, the dry ingredients like wheat flour (130 g), baking soda (1 g), and salt (0.5 g) were added and kneaded by hand into a soft, non-sticky dough, which was allowed to rest for 30 min. For the fermented flour-incorporated biscuits, the control recipe was used as a base, substituting the wheat flour with fermented kidney bean flour at concentrations of 25 %, 50 %, 75 %, and 100 %, designated as B25, B50, B75, and B100, respectively. The dough was rolled out with a cylindrical rolling pin and cut into 4 cm diameter circles using a circular mold. The biscuits were then placed on a butter paper-lined baking tray and baked in a laboratory-scale oven (MBE-201SE-Z, Murni313 Bakery Equipment, Malaysia) at 180 °C for 15 min. After baking, the biscuits were cooled to room temperature and packed in airtight aluminum pouches for further analysis. Each formulation was prepared in triplicate.

**Table 1:** Development of fermented powder-incorporated biscuits.⁎

Ingredients	Sample				
	Control	Fermented flour-incorporated biscuits			
	**B0**	**B25**	**B50**	**B75**	**B100**
Wheat flour: fermented flour (g)	130:0	97.5:32.5	65:65	32.5:97.5	0:130
Butter (g)	65	65	65	65	65
Powdered sugar (g)	70	70	70	70	70
Baking soda (g)	1	1	1	1	1
Common salt (g)	0.5	0.5	0.5	0.5	0.5
Vanilla essence (ml)	5	5	5	5	5

⁎ B0, control biscuits; B25, Biscuits enriched with 25 % fermented kidney bean flour; B50, Biscuits enriched with 50 % fermented kidney bean flour; B75, Biscuits enriched with 75 % fermented kidney bean flour; B100, Biscuits enriched with 100 % fermented kidney bean flour.

#### 2.2.6 Sensory analysis of biscuits

The sensory evaluation of biscuit samples, including B-1 (control), B25, B50, B75, and B100, was conducted using a nine-point hedonic scale. A panel consisting of 30 individuals, evenly split between males and females aged 23 to 45 years, evaluated attributes including taste, appearance, color, aroma, texture, and overall acceptability. To avoid bias, each sample was coded. The evaluation was carried out in the Food Technology and Nutrition Department at Lovely Professional University and received approval from the institutional ethical committee (Ref. No. LPU/CA/023/25/03/12031).

#### 2.2.7 Physicochemical and nutritional evaluation of biscuits

##### 2.2.7.1 Proximate composition

The proximate composition *i.e.*, moisture, ash, crude protein (Micro-Kjeldahl method), crude fat (Soxhlet extraction method), crude fiber (acid-alkali digestion method) of the control (B0), and B25 biscuit was estimated by using standard AOAC (2005). The amount of carbohydrates was calculated by difference method and conversion factors, this method involves subtracting the sum of the other proximate components (moisture, protein, fat, and ash) from the total weight of the sample. The energy content of the control and fermented kidney bean flour at concentrations of 25 % (B25) biscuits was determined using a bomb calorimeter (IK-211, Ikon instruments, New Delhi, India).

##### 2.2.7.2 Physical properties

The physical quality attributes of both the B0 and B25 were assessed in this study. Parameters such as thickness (mm), diameter (mm), weight (g), and spread ratio were determined following the standardized methods described in AACC (2000). To measure the thickness and diameter of the cookies, a digital vernier caliper (Mitutoyo Corporation, Japan) was employed. Additionally, an electronic weighing balance was used to measure the weight of cookies. The spread ratio, which serves as a crucial indicator of cookie quality, was calculated by dividing the diameter of each cookie by its corresponding thickness, according to eq. (5)

(5) $$\text{Speed ratio}=\left(\frac{\text{Diamter of biscuits}\;\left(\mathrm{mm}\right)}{\text{The thickness of biscuits}\;\left(\mathrm{mm}\right)\;}\right)$$

##### 2.2.7.3 Texture analysis

Textural characteristics, specifically hardness, of both B0 and B25 biscuits, were evaluated using a texture analyzer (TA-TX2i, Stable Micro Systems, Godalming, Surrey, UK) following the method outlined by Han and Lee (2021). Briefly, to determine the hardness (kg), a cylindrical probe with a diameter of 36 mm was utilized to compress the samples to 50 % of their initial height. The test was conducted on the second day after baking, at room temperature, with specific parameters set as follows: compression (return to start), a test speed of 1.5 mm/s, strain deformation of 80 %, and a 20-mm limit. The peak force measured during the test represented the hardness of the cookies. Three tests were performed on each batch of cookies, and the average value was reported.

##### 2.2.7.4 *In-vitro* digestibility of biscuits

The *in-vitro* digestion procedure was performed according to the internationally standardized method described by Benhammouche et al. (2021). The procedure consisted of three sequential steps: an initial digestion with α-amylase to stimulate the oral phase, followed by a digestion with pepsin/ HCl to stimulate the gastric phase, and a digestion with bile salts/pancreatin to stimulate intestinal digestion. Briefly, 1 g of each sample was mixed with 4 ml of simulated salivary fluid (SSF), 0.5 ml of α-amylase solution at 1500 U/ml in SSF, 25 μl of 0.3 M CaCl_2,_ and 475 μl of ultrapure water. After 2 min of incubation, the mixture (10 ml) was mixed with 8 ml of simulated gastric fluid (SGF), 5 μl 0.3 M CaCl_2_, at pH 3 adjusted using 1 M HCl/ultrapure water, for a total added volume of 1.395 ml, before adding 0.5 ml of pepsin solution (25,000 U/ml in SGF) The gastric mixture was then incubated for 2 h in a water bath at 37 °C with sufficient mixing. After incubation, 20 ml of gastric mixture was mixed with 85 ml of simulated intestinal fluid (SIF) electrolyte stock solution, 40 μl 0.3 M CaCl_2_, with an adjusted pH to 7 using 1 M NaOH / ultrapure water, for a total added volume of 3.76 ml, before adding 5 ml of pancreatin solution (800 U/ ml in SIF based on trypsin activity), 2.5 ml of bile solution (160 Mm). The intestinal mixture was also incubated at 37 °C. All digestions were run in triplicate; a parallel assay was performed for each sample to determine the amount of acid or base necessary to adjust the digest's pH. Blanks were performed just with enzymes and all samples were blank-corrected. After the simulated gastrointestinal digestion, the samples were cooled by immersion in an ice bath and then centrifuged at 5000 *g* for 10 min at 4 °C to separate the soluble bio-accessible fraction from the residual fraction. Supernatants from the bio-accessible fraction were subsequently frozen at −20 °C for further analysis. Protein digestibility was determined using Eq. (6).

(6) $$\text{Protein digestibility}\;\left(\%\right)=\left[\left(\frac{\text{Bioaccesible protein}\;\left(\mathrm{mg}/g\right)}{\text{Initial protein content}\;\left(\mathrm{mg}/g\right)}\right)\times 100\right]$$

#### 2.2.8 Statistics

Experiments were performed in triplicate, and the results were reported as the mean value with the standard deviation calculated from the three measurements. Statistical evaluation was carried out using one-way analysis of variance (ANOVA) and *t*-tests, with the threshold for statistical significance established at a *p*-value of less than 0.05.

## 3 Results and discussion

### 3.1 Microbial growth

**Fig. 1:**
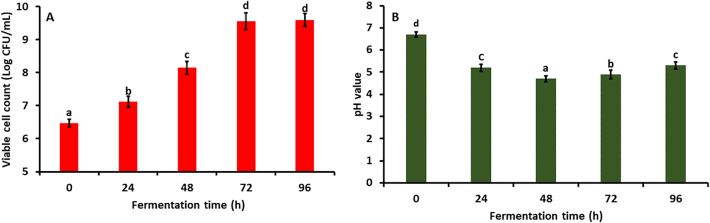
(A). Viable cell count, (B). PH of fermented kidney bean flour at 0 (control), 48, 72, and 96 h using *Aspergillus oryzae* MTCC 548. The results were expressed as the mean ± standard deviation (*n* = 3), and error bars represent the standard deviation from the mean values, while different lowercase letters (a-e) above each bar represent significantly different values within samples based on analysis of variance (ANOVA).

The viable cell count of fermented kidney bean flour using *Aspergillus awamori* MTCC 548 over a duration of 0 to 96 h, as shown in Fig. 1A. Initially, *A. awamori* had a concentration of 6.47 ± 0.12 Log CFU/ml. By 24 h, it significantly enhanced (*p* *<* *0.05*) to 7.12 ± 0.16 Log CFU/ml. During this phase, *A.awamori* likely synthesizes essential enzymes and macromolecules, adjusting to the nutrient conditions of the kidney bean flour substrate (Alae-Carew et al., 2022). There was a significant increase (*p* *<* *0.05*) in growth between 24 and 48 h, reaching 8.15 ± 0.19 Log CFU/ml. The rapid increase in cell count is attributed to the effective nutrient utilization by *A.awamori*, likely availability of abundant amino acids, sugars, and essential nutrients in the substrate, driving exponential growth (Bajić et al., 2022). Furthermore, at 72 h, the concentration was significantly (*p* *<* *0.05*) increased to 9.56 ± 0.25 Log CFU/ml, reflecting continued exponential growth. However, at 96 h, the concentration exhibited a non-significant rise *(p* *>* *0.05)* to 9.60 ± 0.18 Log CFU/ml, suggesting the onset of the stationary phase. This plateau is likely due to nutrient depletion and metabolic waste accumulation. Our findings are in agreement with the results presented by (Y. Liu et al., 2023) as they demonstrate a significant increase in log (cfu/g) values of chickpea flour over fermentation time, reflecting the growth of *lactobacillus plantarum* HLJ29L.

### 3.2 pH determination

As illustrated in Fig. 1B, the pH of *Aspergillus awamori MTCC 548* during a 96-h fermentation shows significant changes, reflecting the fungus's metabolic activities. Initially (0 h), the pH was 6.7 ± 0.12 and reduced significantly (*p* *<* *0.05*) to 5.2 ± 0.16 at 24 h and 4.7 ± 0.14 at 48 h due to organic acid production from substrate breakdown. This acid production results from the metabolic processes where sugars and amino acids are converted into organic acids like citric, gluconic, and oxalic acids, which are common metabolites of *A.awamori* (Behera, 2020). By 72 h, the pH significantly increased (*p* *<* *0.05*) to 4.9 ± 0.19. This increase is due to the medium's buffering capacity or the fungal cells consuming certain acidic metabolites. At 96 h, the pH rises to 5.3 ± 0.15, indicating the transition to the stationary phase with nutrient depletion and waste accumulation, stabilizing metabolic activities. These pH fluctuations align with typical fungal fermentation behavior, where initial acid production lowers pH, followed by stabilization (Qayyum et al., 2024). Our study is similar to the research conducted by (Y. Liu et al., 2023), which shows that significant changes occur in the pH of pea flour as fermentation time increases.

### 3.3 Functional properties

#### 3.3.1 Bulk density

**Fig. 2:**
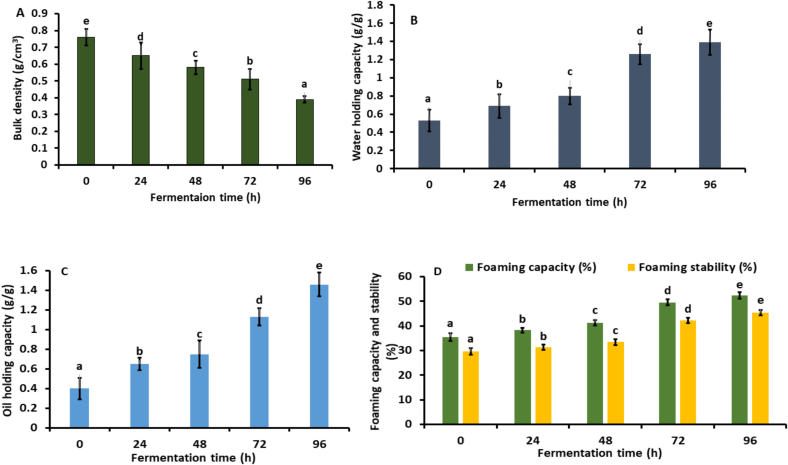

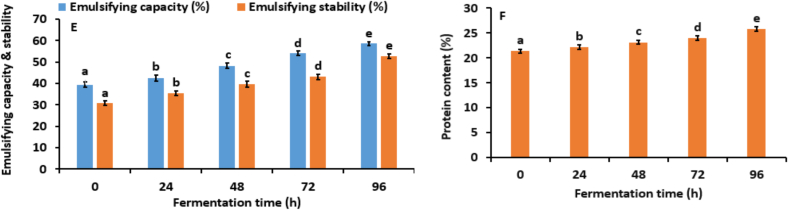
(A). Bulk density, (B).Water holding capacity, (C). Oil holding capacity, (D).Foaming capacity and stability (E). Emulsifying capacity and stability and (F). Protein content of fermented kidney bean flour at 0 (control), 48, 72, and 96 h using *Aspergillus oryzae* MTCC 548. The results were expressed as the mean ± standard deviation (n = 3), and error bars represent the standard deviation from the mean values, while different lowercase letters (a-e) above each bar represent significantly different values within samples based on analysis of variance (ANOVA).

The bulk density of the fermented kidney bean flour using *Aspergillus awamori* MTCC 548 for 0 to 96 h changes significantly as shown in Fig. 2A. Initially the bulk density of the native flour was 0.76 ± 0.05 g/cm^3^. After 24, 48, 72, and 96 h of fermentation, bulk density significantly reduced (*p* *<* *0.05*) to 0.39 ± 0.02 g/cm^3^ in contrast to the native flour. The reduction in bulk density during fermentation can be ascribed to several factors linked to the metabolic processes of *A. awamori*. The breakdown of complex substrates such as carbohydrates and proteins by the fungus to release metabolites and gases (Sara et al., 2021). This decomposition creates voids or air pockets within the substrate matrix, which consequently lowers its bulk density (Nascimento et al., 2022). Our findings are consistent with the study by (Chawla, Chawla, et al., 2017), who reported a significant reduction in the bulk density of fermented black-eyed pea seed flour as fermentation time increased.

#### 3.3.2 Water and oil holding capacity (WHC and OHC)

Fig. 2B illustrates a significant enhancement (*p* *<* *0.05*) in the water holding capacity (WHC) of the fermented kidney bean flour when treated with *Aspergillus awamori* MTCC 548 for 96 h by 61.87 % as compared to non-fermented flour (0 h). This augmentation in WHC can be attributed to the fermentation. During fermentation, the breakdown of complex substrates by microbial activity releases soluble compounds and increases the porosity of the substrate matrix. Consequently, the substrate becomes more capable of retaining water molecules within its structure (Sztupecki et al., 2023). These results align with the research by (Li et al., 2022), which demonstrated that the water-holding capacity of fermented prosomillet bran increases with longer fermentation times when using lactic acid bacteria. Additionally, Fig. 2C demonstrates, that the OHC of the kidney bean flour fermented for 96 h significantly (*p* *<* *0.05*) increased by 72.86 % relative to the OHC of the flour before fermentation. Fermentation improves the oil-holding capacity of flour by facilitating proteolytic activity, which breaks down protein structures, exposing hydrophobic amino acid residues and subsequently increasing the overall hydrophobicity of the flour matrix (Nascimento et al., 2022). Our results correspond with the study by (Kumitch et al., 2020), which reported a 48.01 % increase in the OHC of pea flour fermented with *Aspergillus niger* NRRL 334.

#### 3.3.3 Foaming capacity and stability (FC and FS)

Foaming capacity denotes a flour's ability to produce foam, quantified by the volume of foam generated. Foaming stability is the ability of foam to maintain its structure over time without collapsing. The FC and FS of fermented kidney bean flour (96 h) rise significantly *(p* *<* *0.05)* by 32.30 and 34.81 % as compared to non-fermented (0 h) flour as depicted in Fig. 2D. The improved foaming capacity and stability are due to *A. awamoris* production of proteolytic enzymes during fermentation (Akharume et al., 2021). These enzymes degrade kidney bean proteins into smaller peptides and amino acids, which enhance foam generation and stability (Batbayar, 2022). Our findings are comparable to those of (Borremans et al., 2020), who observed an increase in FC and FS of *Tenebrio molitor* powder fermented with *Lactobacillus curvatus* for 48 h, showing improvements of 70 % and 92 %, respectively.

#### 3.3.4 Emulsifying capacity and stability (EC and ES)

EC is the ability of flours to facilitate the creation of stable oil and water mixtures. ES describes the ability of these mixtures to stay uniform over time without separating. As represented in Fig. 2E, the EC and ES of the kidney bean flour fermented for 96 h significantly increased (*p* *<* *0.05*) by 32.67 % and 47.37 %, respectively, compared to the native flour (0 h). This increase is probably due to the fungus's proteolytic enzymes, which expose hydrophobic regions and shift the hydrophilic-lipophilic balance, thereby improving emulsification (Akharume et al., 2021). Fermentation also produces smaller peptides that enhance emulsification by more effectively interacting with the oil-water interface (Kumar et al., 2022). Our results are consistent with those of (Chawla, Bhandari, et al., 2017), who reported that the emulsifying capacity and stability of fermented black-eyed pea seed flour increased by 33.33 % and 45.58 %, respectively, compared to the non-fermented powder.

#### 3.3.5 Protein content

As shown in Fig. 2F, the Protein content of fermented flour (96 h) significantly increased (*p* *<* *0.05*) by 17.41 % as compared to non-fermented flour. The increase is attributed to the breakdown of structural components like fiber further concentrates the protein, while enzymatic hydrolysis of proteins into peptides and amino acids makes them easier to quantify, contributing to the apparent increase in protein content (Chai et al., 2020; Chawla et al., 2017).

In conclusion, the fermentation of kidney bean flour using *Aspergillus awamori* MTCC 548 significantly enhances its functional properties at 96 h compared to the native flour. Bulk density decreased due to increased porosity from metabolic activities. Water and oil holding capacities improved due to exposed hydrophobic groups. Foaming capacity and stability were enhanced by proteolytic enzyme activity, and emulsifying capacity and stability increased through enzymatic hydrolysis. These findings highlight the effectiveness of 96 h of fermentation in improving the functional properties of kidney bean flour, thereby making it a valuable ingredient for various food applications.

### 3.4 Characterization

#### 3.4.1 Scanning electron microscope (SEM)

**Fig. 3:**
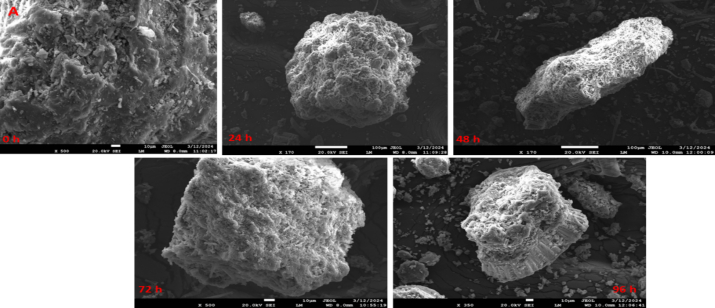

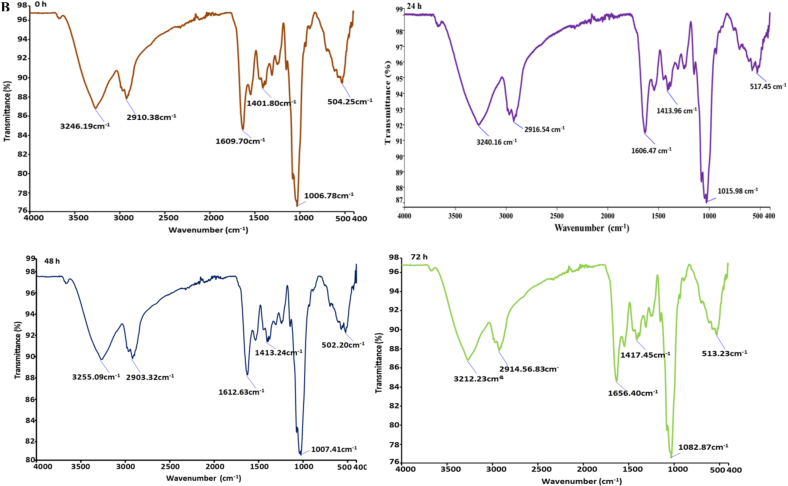

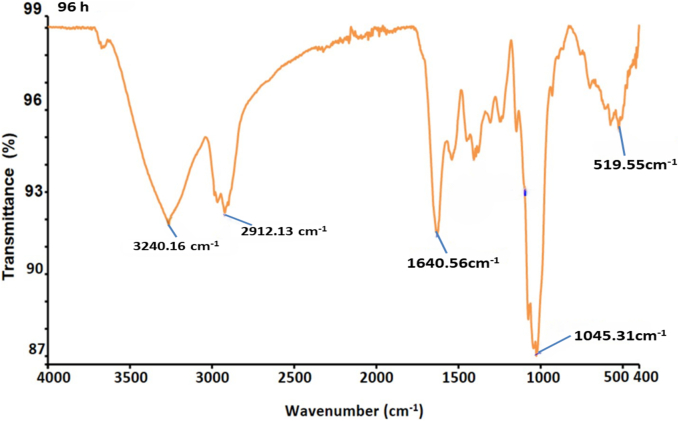

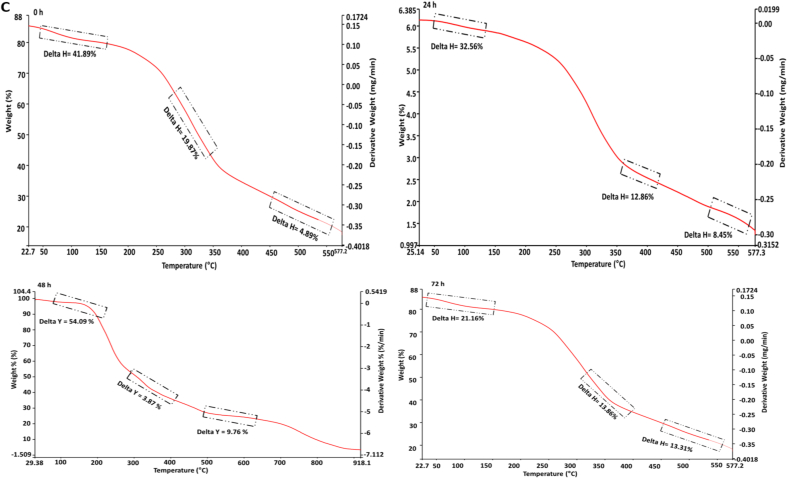

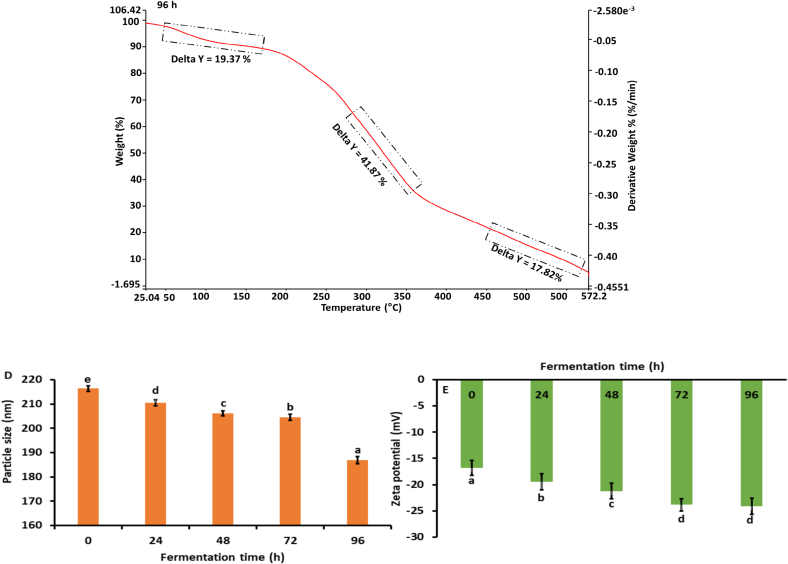
(A). Scanning electron microscope, (B). Fourier transform infrared spectroscopy, (C) Thermogravimetric analysis, (D). Particle size, and (E). Zeta potential of fermented kidney bean flour at 0 (control), 48, 72, and 96 h using *Aspergillus oryzae* MTCC 548. The results were expressed as the mean ± standard deviation (n = 3), and error bars represent the standard deviation from the mean values, while different lowercase letters (a-e) above each bar represent significantly different values within samples based on analysis of variance (ANOVA).

The Scanning Electron Microscopy (SEM) images illustrate the morphological changes in kidney bean flour during fermentation with *Aspergillus awamori* over 0, 24, 48, 72, and 96 h as shown in Fig. 3A. Initially, at 0 h, the untreated flour appears rough and densely packed with irregular, sharp-edged particles. After 24 h, signs of microbial colonization emerge, with slight particle aggregation and increased surface roughness due to initial enzymatic activity. By 48 h, significant changes are evident, with particles clumping into larger aggregates and a more porous surface structure, indicating extensive enzymatic breakdown (Sonta & Rekiel, 2020). At 72 h, the flour shows highly aggregated particles and increased porosity, reflecting advanced degradation and substantial biochemical transformations. After 96 h, the surface becomes highly porous, with extensively fragmented and aggregated particles, suggesting a nearly complete breakdown of the bean matrix. These morphological changes highlight the dynamic nature of fermentation driven by microbial and enzymatic activity. The increasing porosity and fragmentation suggest efficient enzymatic degradation by *Aspergillus awamori*, breaking down complex macromolecules into simpler forms. Active microbial colonization and biofilm formation are crucial for efficient substrate utilization and metabolic interactions (Bhardwaj et al., 2014). Our findings are consistent with those observed in the study by (H. Zhang et al., 2012), which shows that after fermentation the porosity and fragmentation of shrimp shell powder increase.

#### 3.4.2 Fourier transformation infrared spectroscopy (FTIR)

The FTIR analysis of fermented kidney bean flour using *Aspergillus awamori* over different periods (0 h, 24 h, 48 h, 72 h, and 96 h) reveals significant biochemical changes as depicted in Fig. 3B. At 0 h, prominent peaks at 3246.19 cm^−1^ and 2910.38 cm^−1^ indicate the presence of hydroxyl (–OH) groups, suggesting hydrogen bonding in carbohydrate-rich materials like flour, and C—H stretching vibrations from aliphatic compounds, indicating lipid components in kidney bean flour. As fermentation progresses to 24 h, the spectra show shifts in these peaks, reflecting changes in the aliphatic content and the hydrogen bonding environment of hydroxyl groups (Cebrián & Ibarruri, 2023). By 48 h, further shifts in O—H and C—H stretching vibrations are observed, along with consistent N—H bending peaks, indicating ongoing modifications in protein structures. At 72 h, a notable peak at 1656.40 cm^−1^ emerges, suggesting new amide bond formations or significant changes in protein structures, accompanied by changes in polysaccharide content as indicated by the C—O stretching peak at 1082.87 cm^−1^ (Sara et al., 2021). Finally, at 96 h, the spectra show stability in hydroxyl groups with peaks at 3240.16 cm^−1^, but significant changes in protein-related peaks at 1640.56 cm^−1^ and pronounced polysaccharide modifications at 1045.31 cm^−1^. Throughout the fermentation process, the consistent presence and shifting of aliphatic, hydroxyl, and protein-related peaks indicate extensive biochemical interactions between the fungal enzymes and the bean matrix, leading to the formation of new functional groups and the alteration of existing compounds (J. Wang et al., 2023). Our results aligned with the study of (C. Zhang et al., 2020).

#### 3.4.3 Thermogravimetric analysis (TGA)

The thermogravimetric analysis (TGA) of fermented kidney bean flour using *Aspergillus awamori* at various fermentation times (0 h, 24 h, 48 h, 72 h, and 96 h) reveals significant changes in thermal stability and decomposition patterns as represented in Fig. 3C. At 0 h (control), the initial weight loss (41.89 %) between 30 °C and 150 °C is attributed to the moisture and volatile compounds, followed by a notable decomposition (30.17 %) of organic materials between 150 °C and 350 °C, and a final weight loss (8.99 %) of more stable residues from 350 °C to 550 °C. As fermentation progresses to 24 h, the initial weight loss increases to 52.56 %, indicating higher moisture content, while the subsequent decomposition of organic material (12.66 %) and stable residues (8.45 %) remains significant. At 48 h, the moisture content remains high with an initial weight loss of 54.09 %, and the decomposition pattern shows a reduced breakdown of stable residues (3.17 %). By 72 h, there is a decrease in moisture content (21.16 %), but a substantial decomposition (18.18 %) of organic material and higher final weight loss (13.39 %) of stable residues. At 96 h, the moisture content is consistent (19.37 %), with continued decomposition (12.82 %) of organic materials and increased breakdown of stable residues (17.82 %). Fermentation time significantly impacts the thermal stability and composition of kidney bean flour. Extended fermentation (72 h and 96 h) leads to greater decomposition of both volatile and stable organic compounds, reflecting the enzymatic breakdown by *Aspergillus awamori*. Early stages of fermentation show higher initial weight loss due to moisture and volatiles, indicating increased metabolic activity. Substantial decomposition between 150 °C and 350 °C for control and 72 h samples suggests fermentation alters organic material stability. Increased final weight loss at 72 h and 96 h signifies a thorough breakdown of stable components, while shorter fermentation times (0 h and 24 h) maintain greater thermal stability. Our findings are in agreement with the study by (Janiszewska-Turak et al., 2022).

#### 3.4.4 Particle size and zeta potential (PS and ZP)

The particle size and zeta potential of fermented kidney bean flour using *Aspergillus awamori* was measured over a 0 to 96 h fermentation period as shown in Fig. 3D, E and F. The size of the particle significantly reduced (*p* *<* *0.05*) from 216.4 ± 1.18 nm (0 h) to 186.9 ± 1.54 nm (96 h). This decrease is attributed to the enzymatic activity of *Aspergillus awamori*, which breaks down complex macromolecules in the bean flour into simpler molecules. The significant reduction in particle size highlights the efficiency of the fermentation process (Khorsandi, 2022). This enzymatic degradation enhances the solubility, bioavailability, and functional properties of the flour, suggesting its potential for improved applications in food and pharmaceutical industries (K. Liu et al., 2021). Furthermore as represented in Fig. 3E, the negative charge of fermented flour significantly augmented (*p* *<* *0.05*) from −16.84 ± 1.41 to −24.13 ± 1.52 mV at 96 h. The increase in the negative zeta potential suggests enhanced stability of the dispersed particles over time, likely due to the enzymatic activity of *Aspergillus awamori*. The production of organic acids and other charged molecules during fermentation could contribute to the increased surface charge, leading to greater electrostatic repulsion between particles. The observed changes in zeta potential underscore the efficiency of the fermentation process in modifying the surface properties of kidney bean flour (Zaman et al., 2017).

The fermentation of kidney bean flour with *Aspergillus awamori* significantly enhances its morphological, biochemical, thermal, and surface properties over 96 h. SEM analysis shows increased porosity and fragmentation, while FTIR reveals extensive biochemical changes. TGA indicates altered thermal stability and decomposition patterns. Particle size reduction and increased negative zeta potential highlight improved solubility, bioavailability, and stability.

### 3.5 Mineral content of fermented and non-fermented flour

**Fig. 4:**
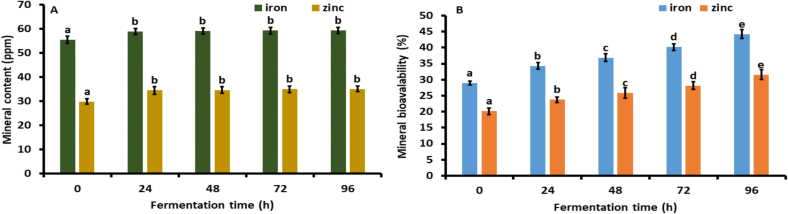
(A). Mineral content, and (B). Mineral bioavailability of fermented kidney bean flour at 0 (control), 48, 72, and 96 h using *Aspergillus oryzae* MTCC 548. The results were expressed as the mean ± standard deviation (n = 3), and error bars represent the standard deviation from the mean values, while different lowercase letters (a-e) above each bar represent significantly different values within samples based on analysis of variance (ANOVA).

As illustrated in Fig. 4A, mineral content of kidney bean flour fermented with *Aspergillus awamori* rose during the fermentation process Specifically, the iron concentration increased significantly (*p* *<* *0.05*) from 55.45 ± 1.45 to 58.96 ± 1.23 ppm after 24 h of fermentation, and after the 24 h of fermentation, it non-significantly (*p* *>* *0.05*) increased by 0.59 % (24 to 96 h). Similarly, the zinc content significantly (*p* *<* *0.05*) rose from 29.86 ± 1.13 to 34.56 ± 1.56 ppm after 24 h. Subsequently, it enhanced non-significantly to 35.13 ± 1.14 ppm at 96 h. The significant rise in mineral content within the first 24 h can be attributed to the enzymatic activities of *Aspergillus awamori*, which likely break down phytic acid, releasing bound minerals and enhancing their bioavailability (Adebo et al., 2022). After the initial 24 h, the augmentation in mineral content becomes non-significant, possibly due to either the saturation of enzyme activity or the exhaustion of substrates that release these minerals (Yoon et al., 2014). Our results are in line with (Chawla et al., 2017), who observed a notable increase in the mineral content of black-eyed peas following solid-state fermentation.

The fermentation of kidney bean flour with *Aspergillus awamori* significantly increases iron and zinc content within the first 24 h, with further increases becoming non-significant. This suggests a shorter fermentation period efficiently enhances mineral bioavailability, optimizing the nutritional quality for food applications.

### 3.6 Mineral bioavailability of fermented and non-fermented flour

The mineral bioavailability of iron and zinc in fermented kidney bean flour (96 h) significantly increased by 34.53 and 36.30 % relative to the non-fermented flour, as illustrated in Fig. 4B. The significant increase in mineral bioavailability after fermentation of kidney bean flour using *Aspergillus awamori* can be attributed to several biochemical and enzymatic changes. During fermentation, *Aspergillus awamori* produces phytase enzymes that degrade phytic acid, a common anti-nutritional factor in legumes that binds minerals like iron and zinc, forming insoluble complexes. The breakdown of phytic acid releases these bound minerals, making them more bioavailable (Adebo et al., 2022). Furthermore, the fermentation process results in acidification as a consequence of organic acid production, which can solubilize minerals and enhance their absorption. Enzymes such as amylase, protease, and cellulase produced during fermentation also degrade other anti-nutritional factors like tannins and oxalates, further improving mineral availability (Chawla, Chawla, et al., 2017; Suiryanrayna & Ramana, 2015). The metabolic activities of *A. awamori* generate metabolites that enhance mineral solubility, while the physical breakdown of the bean matrix by these enzymes releases minerals trapped within the cellular structures (Mrinal et al., 2021). Collectively, these processes release bound minerals and enhance their solubility and availability for absorption during digestion, significantly increasing mineral bioavailability. Our results are in line with Chawla, et al., (2017), which demonstrated a significant increase in the mineral bioavailability of black-eyed peas following solid-state fermentation.

Based on the assessment of physicochemical and functional properties, and mineral bioavailability, the 96 h fermented kidney bean flour was selected for the application and further analysis.

### 3.7 Sensory analysis of biscuit

As presented in Table 2, the sensory evaluation of biscuit samples (B0, B25, B50, B75, and B100) demonstrated significant variations in color and appearance, aroma, taste, texture, and overall acceptability. The color and appearance scores decreased significantly (*p* *<* *0.05*) from B0 (8.01 ± 0.71) to B100 (4.78 ± 0.18), with B25 (7.95 ± 0.51) showing a non-significant (*p* *>* *0.05*) difference from the control (B0), indicating that up to 25 % incorporation of the novel ingredient did not negatively impact the visual appeal. A similar trend was observed for aroma, where B0 (7.77 ± 0.25) and B25 (7.78 ± 0.17) achieved the highest scores, while B100 exhibited the lowest score (5.08 ± 0.18), reflecting reduced aromatic acceptability at higher incorporation levels. For taste, B25 (7.96 ± 0.25) closely matched B0 (8.0 ± 0.84), while further incorporation resulted in reduced scores, with B100 scoring the lowest (4.85 ± 0.36), likely due to an overpowering influence of the additional ingredient. Textural analysis revealed that B0 (7.95 ± 0.78) and B25 (7.79 ± 0.35) maintained favorable scores, while B100 (5.18 ± 0.21) showed a significant decline (*p* *<* *0.05*) in textural quality. Overall acceptability followed a comparable pattern, with B0 (8.08 ± 0.74) and B25 (7.97 ± 1.03) being the most preferred samples, whereas B100 (4.75 ± 0.41) was the least acceptable. These results suggest that B25, with 25 % incorporation, maintains sensory attributes similar to the control and is therefore identified as the most suitable formulation for further investigation.

**Table 2:** Sensory evaluation of control and Fermented kidney bean flour incorporated biscuits.⁎

Samples	Color and appearance	Aroma	Taste	Texture	Overall acceptability
B0	8.01 ± 0.71^d^	7.77 ± 0.25^d^	8.0 ± 0.84^d^	7.95 ± 0.78^c^	8.08 ± 0.74^c^
B25	7.95 ± 0.51^d^	7.78 ± 0.17^d^	7.96 ± 0.25^d^	7.79 ± 0.35^c^	7.97 ± 1.03^c^
B50	7.55 ± 0.16^c^	7.35 ± 0.11^c^	7.41 ± 0.11^c^	7.71 ± 0.88^c^	7.75 ± 0.91^c^
B75	6.08 ± 0.54^b^	6.05 ± 0.19^b^	6.05 ± 0.59^b^	6.31 ± 0.37^b^	5.98 ± 1.01^b^
B100	4.78 ± 0.18^a^	5.08 ± 0.18^a^	4.85 ± 0.36^a^	5.18 ± 0.21^a^	4.75 ± 0.41^a^

⁎ Results are expressed as mean ± standard deviation (*n* = 30). The mean values with lowercase superscripts (a-d) within the column represent statistically significant differences (*p* *<* *0.05*) based on ANOVA and post-hoc Duncan's multiple range tests. B0, control biscuits; B25, Biscuits enriched with 25 % fermented kidney bean flour; B50, Biscuits enriched with 50 % fermented kidney bean flour; B75, Biscuits enriched with 75 % fermented kidney bean flour; B100, Biscuits enriched with 100 % fermented kidney bean flour.

### 3.8 Proximate composition of biscuits

As represented in Table 3, the proximate composition of the control biscuits (B0) and fermented flour-incorporated biscuits (B25) exhibited both significant and non-significant differences across various parameters. The crude protein content was significantly higher (*p* *<* *0.05*) in B25 (19.8 ± 0.74 %) compared to B0 (12.7 ± 0.09 %), which can be attributed to the fermentation process. Fermentation likely improved protein availability by reducing anti-nutritional factors and enhancing the digestibility of proteins. In contrast, the crude fat content showed a significant (*p* *<* *0.05*) reduction in B25 (22.4 ± 0.16 %) compared to B0 (24.6 ± 0.11 %), which may be due to the microbial metabolism of lipids during fermentation, resulting in lower residual fat in the fermented flour. The crude fiber content also exhibited a significant increase (*p* *<* *0.05*) in B25 (2.3 ± 0.09 %) relative to B0 (2.0 ± 0.07 %), suggesting that fermentation may have contributed to the release of bound dietary fiber or modification of cell wall components. Total carbohydrate content showed a significant difference (*p* *<* *0.05*) between B0 (58.56 ± 0.17 %) and B25 (55.6 ± 0.25 %). Furthermore, moisture content also showed a non-significant (*p* *>* *0.05)* difference, with B0 (1.98 ± 0.21 %) and B25 (2.01 ± 0.16 %), suggesting that the incorporation of fermented flour did not substantially alter water retention in the biscuits. Ash content demonstrated a significant increase (*p* *<* *0.05*), with B25 (3.4 ± 0.10 %) slightly higher than B0 (2.16 ± 0.11 %). This increase might be related to the concentration of minerals during the fermentation process. Furthermore, the energy content was significantly (*p* *<* *0.05*) lower in B25 (500.4 ± 1.21 Kcal/g) compared to B0 (532.2 ± 1.15 Kcal/g), primarily due to the reduced crude fat content in B25, as fat is a major contributor to the caloric value of food products. Overall, these results demonstrate that the incorporation of fermented flour significantly improved the protein and fiber content while maintaining carbohydrate, moisture, and ash levels comparable to the control. The lower energy content of B25 further supports its potential as a nutritionally enhanced and functional food product.

**Table 3:** Physio-chemical analysis of biscuits.⁎

Parameters	Control (B0)	Fermented powder incorporated biscuits (B25)
Crude protein (%)	12.7 ± 0.09^a^	19.8 ± 0.74^b^
Crude fat (%)	24.6 ± 0.11^b^	22.4 ± 0.16^a^
Crude fiber (%)	2.0 ± 0.07^a^	2.3 ± 0.09^b^
Total carbohydrate (%)	58.56 ± 0.17^b^	55.6 ± 0.25^a^
Moisture (%)	1.98 ± 0.21^a^	2.01 ± 0.16^a^
Ash (%)	2.16 ± 0.11^a^	3.4 ± 0.10^b^
Energy (Kcal/g)	532.2 ± 1.15^b^	500.4 ± 1.21^a^
Weight (g)	10.21 ± 0.41^a^	11.45 ± 0.31^b^
Diameter (mm)	49.10 ± 0.52^a^	49.56 ± 0.29^a^
Thickness (mm)	10.89 ± 0.16^a^	11.42 ± 0.31^b^
Spread ratio	4.50 ± 0.20^a^	4.41 ± 0.18^a^
Hardness (Kg)	2.68 ± 0.14^a^	2.96 ± 0.11^b^

⁎ Results are expressed as mean ± standard deviation (n = 3). The mean values with lowercase superscripts (a-b) within the column represent statistically significant differences (*p<0.05*) based on t-test. B0, control biscuits; B25, Biscuits enriched with 25 % fermented kidney bean flour; B50, Biscuits enriched with 50 % fermented kidney bean flour; B75, Biscuits enriched with 75 % fermented kidney bean flour; B100, Biscuits enriched with 100 % fermented kidney bean flour.

### 3.9 Physical properties of biscuits

As represented in Table 3, the physical properties of the control biscuits (B0) and the fermented flour-incorporated biscuits (B25) exhibited both significant and non-significant differences. The weight of B25 (11.45 ± 0.31 g) was significantly (*p* *<* *0.05*) higher than that of B0 (10.21 ± 0.41 g), which can be attributed to the addition of fermented flour, potentially increasing the density and mass of the biscuit matrix. Conversely, as shown in Fig. 5 A, the diameter of B25 (49.56 ± 0.29 mm) was non-significantly (*p* *>* *0.05)* higher than B0 (49.10 ± 0.52 mm), indicating a slight increase in dough spread during baking due to the modified rheological properties imparted by the fermented flour. Similarly as shown in Fig. 5B the thickness of B25 (11.42 ± 0.31 mm) was significantly (*p* *<* *0.05*) greater than that of B0 (10.89 ± 0.16 mm), likely due to increased water retention or structural integrity from the fermentation process, which could have enhanced the dough's ability to retain its shape. This resulted in a lower spread ratio for B25 (4.41 ± 0.18) compared to B0 (4.50 ± 0.20), reflecting the interplay between reduced diameter and increased thickness. Hardness, measured as the force required to break the biscuits, was significantly (*p* *<* *0.05*) higher in B25 (2.96 ± 0.11 Kg) compared to B0 (2.68 ± 0.14 Kg). The increased hardness may be attributed to the structural support provided by the fermented flour, which could have contributed to a firmer texture. In conclusion, the incorporation of fermented flour significantly improved the nutritional profile and altered the physical properties of biscuits.

### 3.10 *in-vitro* digestibility of biscuits

As shown in Table 4, the *in-vitro* digestibility of sample B25 significantly (*p* *<* *0.05*) improved by 66.79 % compared to the control sample. This enhancement can be ascribed to the fermentation process using *Aspergillus awamori*. Fermentation improves protein digestibility by lowering anti-nutritional factors such as phytates and tannins, which generally impede protein absorption (Adebo et al., 2022; Chawla, Bhandari, et al., 2017). Additionally, the fermentation process generates enzymes that partially hydrolyze complex proteins and carbohydrates, making them easier to digest (Suiryanrayna & Ramana, 2015). The structural modifications in proteins during fermentation also expose more sites for digestive enzymes to act upon, further improving digestibility (Mrinal et al., 2021). These combined effects led to the significant improvement observed in the B25 sample's *in-vitro* digestibility.

**Table 4:** *In-vitro* digestibility of B0 (Control biscuits) and B25 (fermented kidney bean flour powder at concentrations of 50 %).⁎

Sample	Initial protein content (mg/g)	Bio-accessible protein (mg/g)	Protein digestibility (%)
B0	127.40 ± 0.96^a^	59.93 ± 0.76 ^a^	47.04 ± 0.9 ^a^
B25	152.23 ± 1.56^b^	119.44 ± 1.12 ^b^	78.40 ± 1.09 ^b^

⁎ Values presented as mean ± standard deviation (n = 3). Different letters in the same column show significant differences (*p<0.05*) between B0 (Control biscuits) and B25 (fermented kidney bean flour powder at concentrations of 25 %), from the *t*-test.

## 4 Conclusion

Solid-state fermentation with *Aspergillus awamori* MTCC 548 significantly enhances the structural features, functional properties, and mineral bioavailability of kidney bean flour. Significant improvements include higher foaming and emulsifying properties, oil and water holding capacities, and improved thermal stability, achieved with shorter fermentation durations. The process also significantly boosts iron and zinc content and bioavailability. For future research, exploring product formulation using the enhanced kidney bean flour and conducting *in vivo* studies to assess its nutritional and functional benefits in real-world applications are recommended. These steps will further validate its potential as a nutritionally enriched and functionally superior food ingredient.

## CRediT authorship contribution statement

**Nikhil Dnyaneshwar Patil:** Writing – original draft, Project administration, Methodology, Investigation, Formal analysis. **Aarti Bains:** Writing – review & editing, Methodology. **Gulden Goksen:** Writing – review & editing, Methodology. **Nemat Ali:** Writing – review & editing, Methodology. **Sanju Bala Dhull:** Writing – review & editing, Methodology. **Mohammad Rashid Khan:** Writing – review & editing, Methodology. **Prince Chawla:** Writing – review & editing, Visualization, Validation, Supervision, Project administration, Methodology, Conceptualization.

## Declaration of competing interest

The authors declare that they have no known competing financial interests or personal relationships that could have appeared to influence the work reported in this paper.

## Data Availability

Data will be made available on request.

## Appendix A Supplementary data

Supplementary material Supplementary Figure 3F. Particle size of fermented kidney bean flour at 0 h (control), 48 h, 72 h, and 96 h using *Aspergillus oryzae* MTCC 548. Results are expressed as mean ± standard deviation (n = 3), with error bars representing the standard deviation from the mean values. Supplementary Figure 5(A). Biscuit formulation with varying concentrations of wheat flour and fermented (96 h) kidney bean flour. B0: control biscuits; B25: biscuits enriched with 25% fermented kidney bean flour; B50: biscuits enriched with 50% fermented kidney bean flour; B75: biscuits enriched with 75% fermented kidney bean flour; B100: biscuits enriched with 100% fermented kidney bean flour. and (B) Thickness of B0 and B25 biscuits. Image 1
