# Supplementary material for: Effect of solid-state fermentation on kidney bean flour: Functional properties, mineral bioavailability, and product formulation
Source: Food Chem X. 2025 Mar 6;27:102339. doi: 10.1016/j.fochx.2025.102339 (PMC11952869; doi:10.1016/j.fochx.2025.102339)
Supplement: Supplementary file 1 — Supplementary material Supplementary Figure 3F. Particle size of fermented kidney bean flour at 0 h (control), 48 h, 72 h, and 96 h using Aspergillus oryzae MTCC 548. Results are expressed as mean ± standard deviation (n = 3), with error bars representing the standard deviation from the mean values. Supplementary Figure 5(A). Biscuit formulation with varying concentrations of wheat flour and fermented (96 h) kidney bean flour. B0: control biscuits; B25: biscuits enriched with 25% fermented kidney bean flour; B50: biscuits enriched with 50% fermented kidney bean flour; B75: biscuits enriched with 75% fermented kidney bean flour; B100: biscuits enriched with 100% fermented kidney bean flour. and (B) Thickness of B0 and B25 biscuits. [file mmc1.docx]

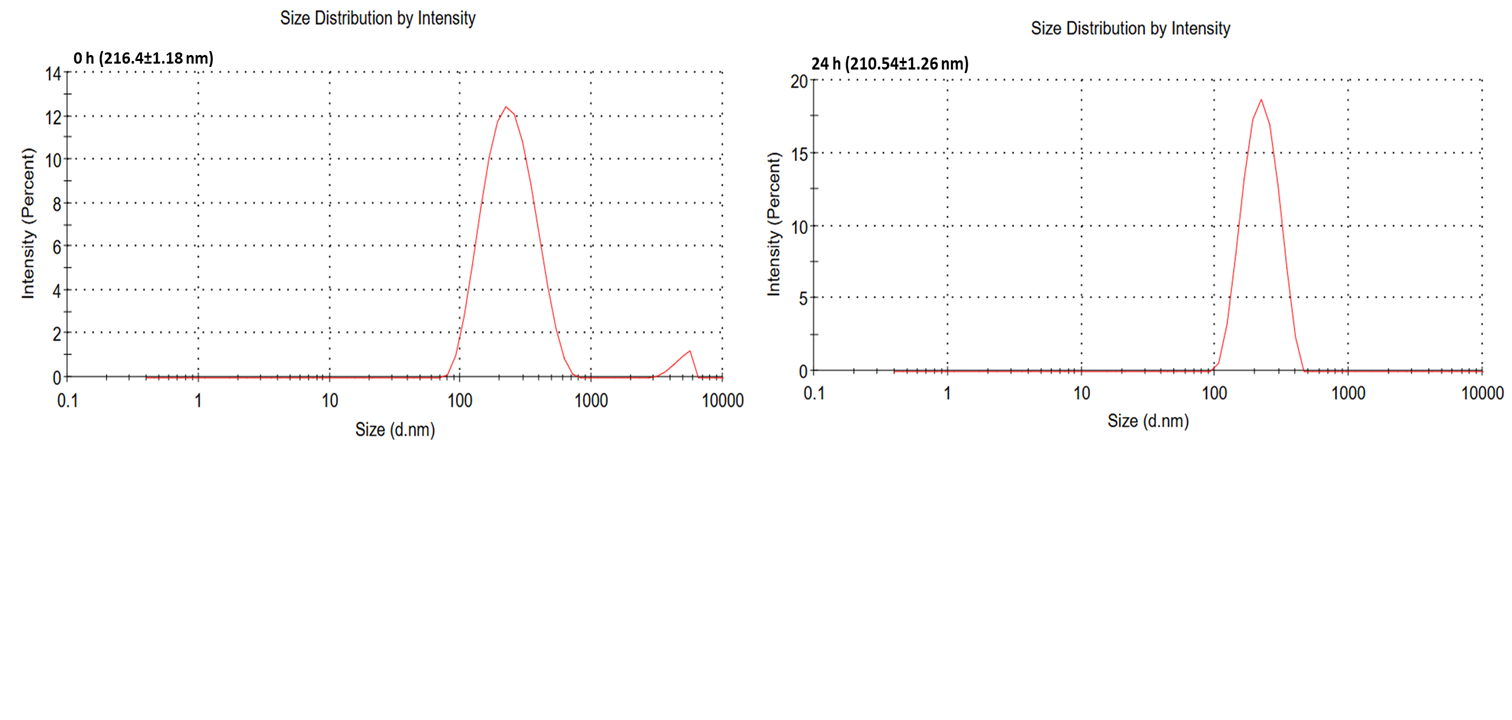

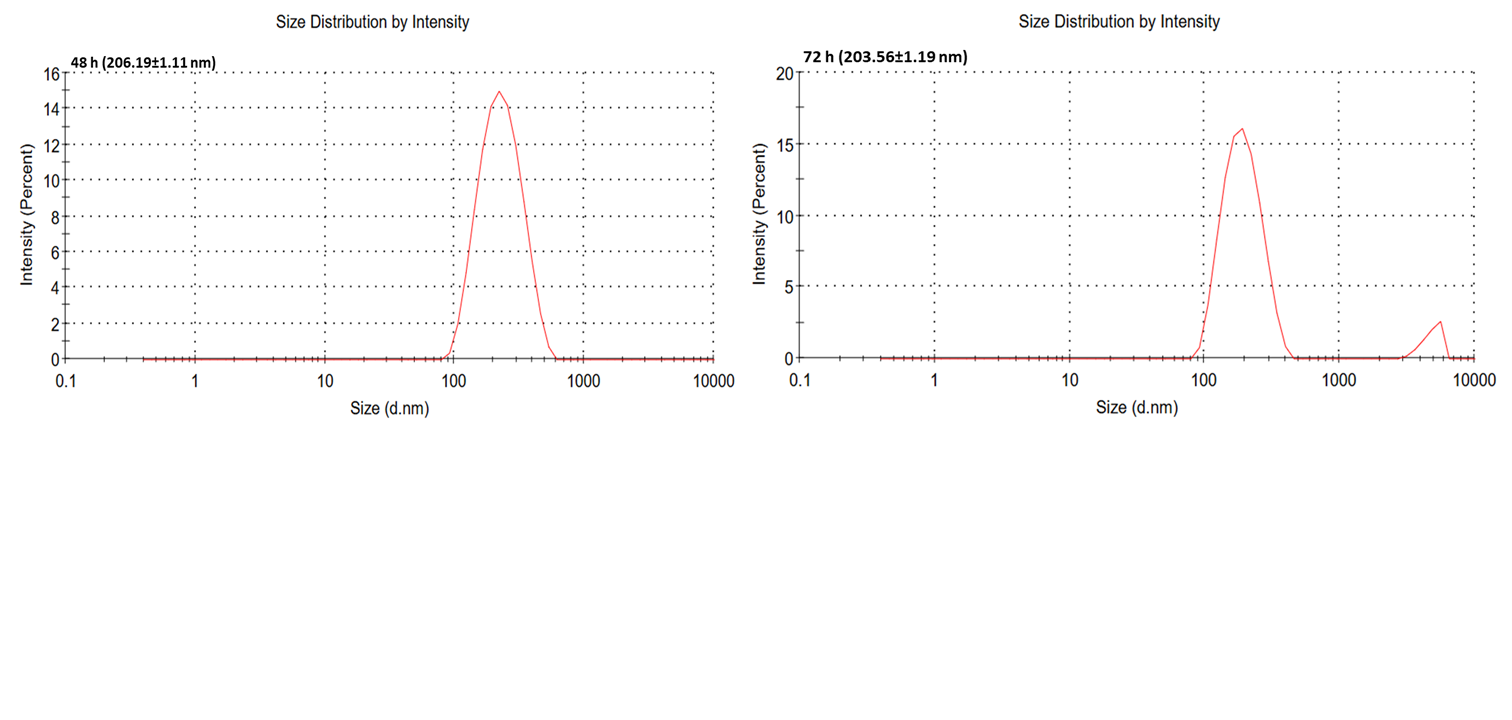


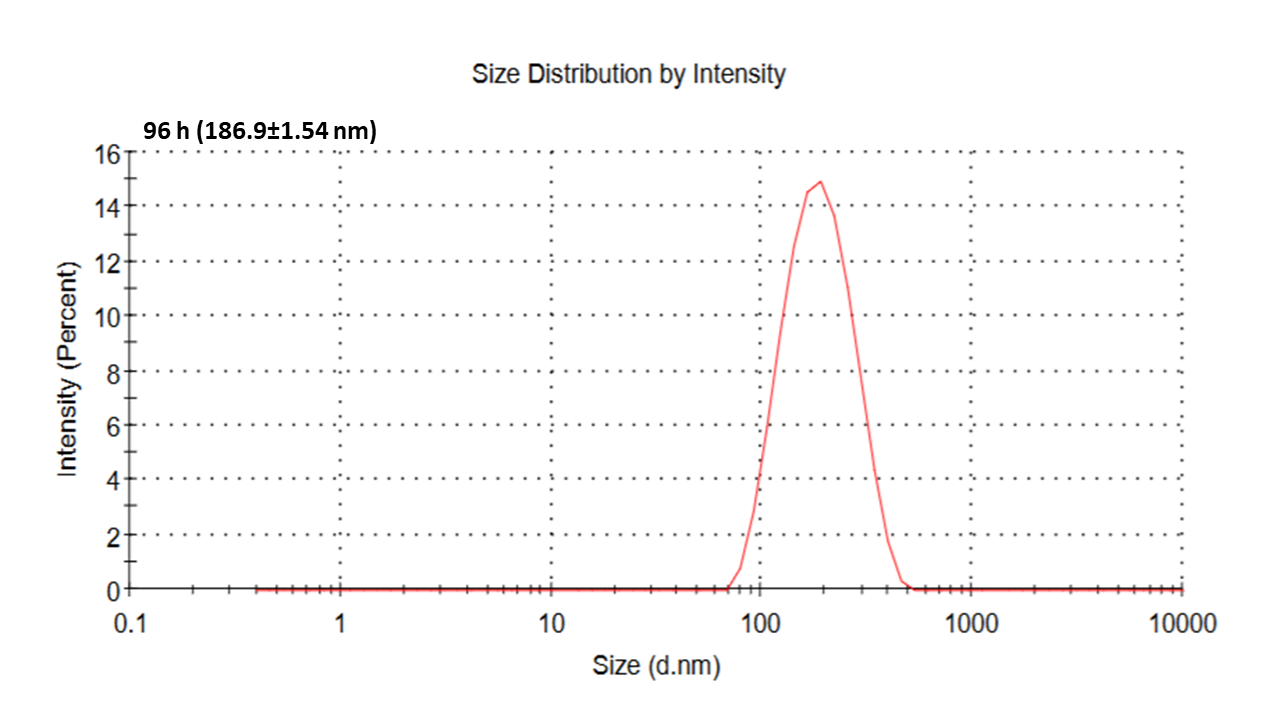


**Fig.3F Particle size of** fermented kidney bean flour at 0 (control), 48, 72, and 96 h using *Aspergillus oryzae* MTCC 548. The results were expressed as the mean ± standard deviation (n = 3), and error bars represent the standard deviation from the mean values, while different lowercase letters above each bar represent significantly different values within samples based on analysis of variance (ANOVA).


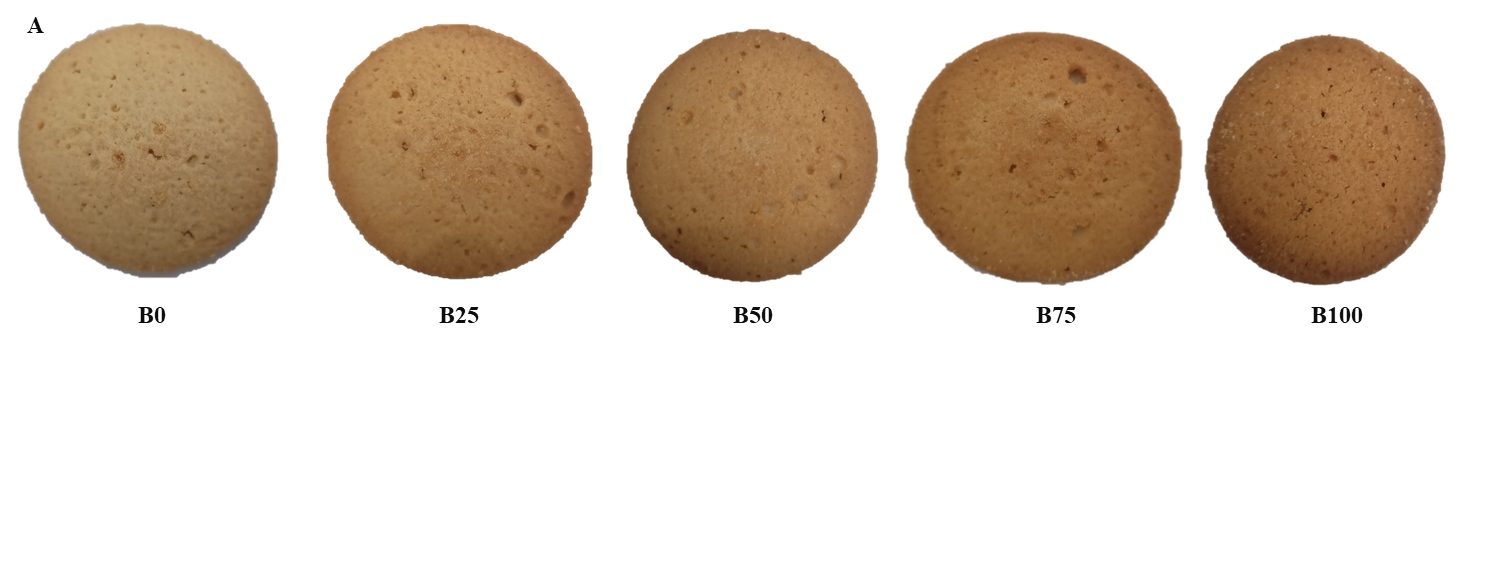


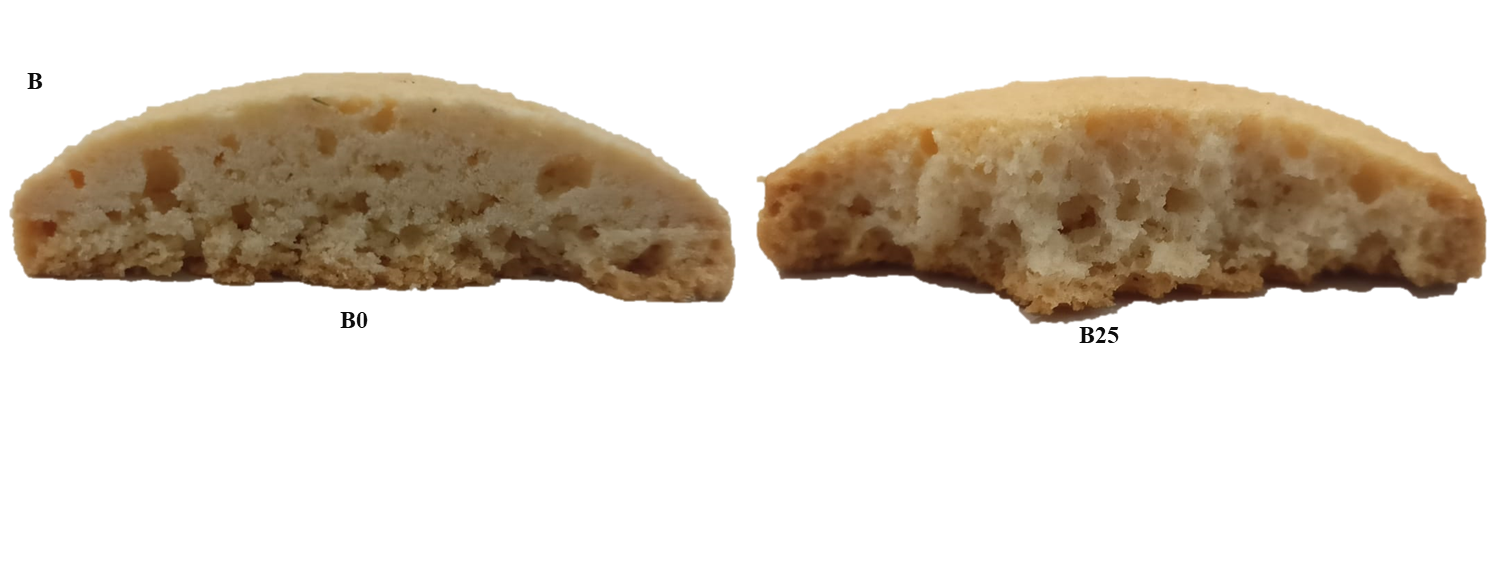


Figure 5A. The biscuit formulation at different concentrations of wheat and fermented (96 h) kidney bean flour. B0, control biscuits; B25, Biscuits enriched with 25 % fermented kidney bean flour; B50, Biscuits enriched with 50% fermented kidney bean flour; B75, Biscuits enriched with 75% fermented kidney bean flour; B100, Biscuits enriched with 100% fermented kidney bean flour and (B). Thickness of the B0 and B25 biscuits. B0, control biscuits; B25, Biscuits enriched with 25 % fermented kidney bean flour
